# Engineering Chondroitinase‐Free Baculovirus‐Insect Cell Expression System for Efficient Synthesis of Chondroitin Sulphates

**DOI:** 10.1111/1751-7915.70365

**Published:** 2026-05-15

**Authors:** Junyue Li, Yuqing Tian, Lihe She, Zhangliang Liu, Yingying Zhou, Yanyan Wang, Jihui Zhang, Leilei Zhu, Huarong Tan, Jine Li

**Affiliations:** ^1^ State Key Laboratory of Microbial Diversity and Innovative Utilization, Institute of Microbiology Chinese Academy of Sciences Beijing China; ^2^ College of Life Sciences University of Chinese Academy of Sciences Beijing China; ^3^ State Key Laboratory of Engineering Biology for Low‐Carbon Manufacturing, Tianjin Institute of Industrial Biotechnology Chinese Academy of Sciences Tianjin China

**Keywords:** chondroitin sulphate, chondroitin sulphotransferases, engineered baculovirus, enzymatic synthesis

## Abstract

Owing to the high expression levels, proper protein folding and post‐translational modifications, baculovirus‐insect cell expression system is an ideal platform for producing active chondroitin sulfotransferases, which specifically catalyse the synthesis of chondroitin sulphate (CS). However, the CS can be degraded by the endogenous chondroitinase derived from the viral envelope protein ODV‐E66, thus requiring elaborate purification, which is impractical for large‐scale production. To address this issue, a chondroitinase‐free baculovirus was constructed by deleting the *odv‐e66* gene from bacterial artificial chromosomes (BACs). Through the engineered baculovirus, highly active chondroitin sulfotransferases (CS‐4OST, CS‐6OST and GalNAc4S‐6OST) were successfully expressed and secreted into the culture supernatant. Of note, the culture supernatant can be directly employed for CS synthesis. Using the cultures harbouring the corresponding sulfotransferases, CS‐A, CS‐C and CS‐E were successfully prepared at the gram‐scale, thus obviating the need for laborious and costly purification. Collectively, this work overcomes the inherent limitation of CS degradation in baculovirus‐insect cell expression system, thereby providing a robust and reliable platform for the expression of chondroitin sulfotransferases.

## Graphical abstract

An engineered baculovirus‐insect cell platform produces chondroitinase‐free sulfotransferases, enabling efficient synthesis of chondroitin sulphates.

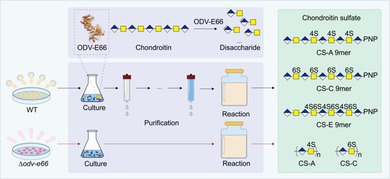

## 1 Introduction

Chondroitin sulphate (CS) is a kind of linear polysaccharide composed of alternating β‐D‐(1 → 3)‐glucuronic acid (GlcA) and β‐D‐(1 → 4)‐N‐acetyl‐galactosamine (GalNAc) residues, which could be modified by sulfation and epimerization to give rise to various CS subtypes with distinct biological activities (Ji et al. 2020). As a major component of extracellular matrix, CS is widely present in animal connective tissues to play critical roles in numerous biological functions, including signal transduction, cell migration, immune cell interaction and so on (Mikami and Kitagawa 2023). Due to its potent anti‐inflammatory activity, CS has been widely used as dietary supplements or pharmaceutical drugs for the treatment of osteoarthritis.

Currently, the commercially available CS is extracted from animal tissues, such as bovine trachea and shark cartilage. However, with the aging of global population, the supply of CS is struggling to meet the growing demand due to the limited raw materials availability and risks of virus or antibiotics contamination (Li et al. 2025). Therefore, innovative and scalable production strategies, particularly those utilizing renewable materials, are urgently needed. Various approaches have been developed to date, including chemical synthesis, enzymatic synthesis and microbial cell‐based production (Ji et al. 2020; Badri et al. 2021; Xiong et al. 2025). Although chemical synthesis enables the preparation of diverse structures (Mende et al. 2016), its poor stereoselectivity usually leads to low efficiency, hindering large‐scale application. In contrast, enzymatic synthesis offers high selectivity and efficiency, rendering it more suitable for CS production (Zhang et al. 2020). Nevertheless, obtaining chondroitin sulfotransferases with high activity and low cost remains challenging. This is because most chondroitin sulfotransferases are of animal origin and typically carry N‐glycosylation modifications. As the modification is critical for enzyme activity or stability (Yusa et al. 2005, 2006), the absence of N‐glycosylation in prokaryotic expression systems impedes the development of both enzymatic and microbial synthesis. Therefore, developing an efficient expression system for chondroitin sulfotransferases with appropriate N‐glycosylation holds great significance for advancing the production of animal‐free chondroitin sulphates.

Baculovirus‐insect cell expression system is a widely used eukaryotic expression platform which delivers exogenous genes into insect cells via recombinant baculovirus for protein production. Owing to its high expression efficiency, ability to mediate sophisticated post‐translational modifications (e.g., glycosylation), and relatively low costs, this system presents unique advantages for the expression of animal‐derived proteins. Among these, the Bac‐to‐Bac baculovirus expression system has been employed to produce recombinant chondroitin 4‐*O*‐sulfotransferase (CS‐4OST), 6‐*O*‐sulfotransferase (CS‐6OST) and 4‐sulphate 6‐*O*‐sulfotransferase (GalNAc4S‐6OST) (Li et al. 2017). These enzymes, which catalyse the sulfation of 4‐OH or 6‐OH on GalNAc residues, have been expressed and employed for the enzymatic synthesis of structurally homogeneous CS oligosaccharides (Li et al. 2017, 2020). However, baculoviruses inherently express a truncated form of occlusion‐derived virus envelope protein 66 (ODV‐E66). As a virus‐specific protein that mediates oral infection of host insect midgut epithelial cells in nature, ODV‐E66 possesses intrinsic chondroitinase activity, which leads to severe degradation of CS (Sugiura et al. 2011). Consequently, elaborate purification of the recombinant sulfotransferases is required to eliminate chondroitinase contamination before being applied to CS synthesis. However, it is impractical in large‐scale production due to the low efficiency and high cost.

To overcome the limitation, we constructed an engineered baculovirus lacking the ODV‐E66 coding gene, thereby eliminating the endogenous chondroitinase expression. Using this engineered baculovirus, CS‐4OST, CS‐6OST and GalNAc4S‐6OST were robustly produced in *Sf9* insect cells. Notably, the resulting cell culture supernatant can be directly employed for CS synthesis, avoiding laborious and time‐consuming protein purification steps, and thus enabling efficient preparation of structurally defined CS.

## 2 Materials and Methods

### 2.1 Construction of the Bacmid‐Δ*odv*‐*e66* Recombinant Plasmid

To delete the *odv‐e66* gene on the baculovirus genome, the bacmid (bMON14272) containing baculovirus DNA and the helper plasmid pIJ790 were successively introduced into *E. coli* BW25113 by electroporation. The resulting recombinant strain, BW25113/Bacmid/pIJ790, was cultured at 30°C on LB agar containing kanamycin (50 μg/mL) and chloramphenicol (25 μg/mL). For recombinant virus construction, the *odv‐e66* gene was replaced with an apramycin resistance cassette via λ‐Red‐mediated recombination. Specifically, the *aac(3)IV* gene was amplified from plasmid pIJ773 (Gust et al. 2003) using primers Ac‐odv‐de66F/Ac‐odv‐de66R (Table S1), which contain 39‐bp homology arms targeting *odv‐e66*. The PCR product was then introduced into the competent cell of BW25113/Bacmid/pIJ790 via electro‐transformation. Transformants were selected on LB plates containing kanamycin (50 μg/mL) and apramycin (100 μg/mL). The resultant plasmid, designated as Bacmid‐Δ*odv‐e66*, was isolated and subsequently transformed into BW25113 cells to eliminate potential contamination from wildtype Bacmid. Successful replacement of the *odv‐e66* gene with the *aac(3)IV* cassette was verified by PCR using primers Ac‐odv‐de66F/Ac‐odv‐de66R and e66check‐F/R (Table S1), as well as DNA sequencing.

### 2.2 Expression of Recombinant Protein

The plasmids pFastBac‐Mel‐HT‐CS4OST, pFastBac‐Mel‐HT‐CS6OST and pFastBac‐Mel‐HT‐CS4S6OST (Li et al. 2017, 2020), were introduced into DH10/Bacmid‐Δ*odv‐e66* competent cells harbouring the helper plasmid pMON7124. Transformants were selected on LB agar plates supplemented with kanamycin (50 μg/mL), gentamicin (7 μg/mL), tetracycline (10 μg/mL), X‐gal (40 μg/mL) and IPTG (40 μg/mL). White colonies, indicative of successful transposition, were picked. The resulting recombinant bacmids were then used to generate the corresponding baculoviruses Δ*odv‐e66*‐CS‐4OST, Δ*odv‐e66*‐CS‐6OST and Δ*odv‐e66*‐GalNAc4S‐6OST by the Cellfectin II Reagent (Thermo Fisher Scientific) following the manufacturer's instructions. *Sf9* cells were cultured in SIM SF medium with 0.5 × 10^6^ cells/mL and incubated at 130 rpm with shaking at 28°C. Transfection was performed when the cell concentration reached 2.0 × 10^6^ cells/mL. After 3–4 days of culture, the supernatant was collected by centrifugation at 500 × g for 5 min at 4°C. Glycerol (20% v/v), Triton X‐100 (0.1% v/v) and PMSF (1 mM) were then added in and mixed. The resulting crude enzyme solution was subjected to activity and kinetic assays, or stored at −20°C for subsequent synthesis experiments.

### 2.3 Western Blot Analysis

The recombinant proteins were separated by 10% SDS‐PAGE and transferred onto PVDF membranes using a wet transfer system (Mini Trans‐Blot) in transfer buffer (25 mM Tris‐base, 200 mM glycine, 0.1% SDS, 20% methanol, pH 8.3). Membranes were blocked with 5% non‐fat dry milk in TBST (20 mM Tris–HCl pH 7.6, 150 mM NaCl, 0.1% Tween‐20) for 1 h at room temperature, then incubated overnight at 4°C with primary antibody (anti‐His‐Tag, rabbit polyclonal antibody, purchased from Easybio). After washing three times with TBST, membranes were incubated with horseradish peroxidase (HRP)‐conjugated secondary antibody (Easybio) for 1 h at room temperature. Signals were detected using an ECL chemiluminescence kit and visualized via a ChemiDoc Imaging System (Tanon 5200 Multi).

### 2.4 Deglycosylation Analysis

To analyse N‐glycosylation, protein samples were treated with PNGase F using a commercial deglycosylation kit (Beyotime Biotechnology) following the manufacturer's protocol. Briefly, proteins were incubated with PNGase F at 37°C for 1 h, followed by Western blot analysis as described in section 2.3. After incubation, the reaction mixture was used directly for the subsequent activity assays without further purification. Enzymatic activities of the three sulfotransferases were determined using oligosaccharides as the substrates. Product formation was analysed by HPLC at a wavelength of 310 nm using a ProPac PA1 column (9 × 250 mm; Thermo Fisher Scientific). Mobile phases consisted of (A) 20 mM sodium acetate (pH 5.0) and (B) 20 mM sodium acetate containing 1 M sodium chloride (pH 5.0). The flow rate was 1 mL/min, and a linear gradient from 0% to 100% B over 40 min was applied for elution.

### 2.5 Enzyme Property Assay

Activity assays were performed in a 1 mL reaction mixture containing 3 mg chondroitin, 16 μmol PAPS, 50 mM MES buffer (pH 6.2), 2 mM CaCl_2_ and 50 μL of crude enzyme extract. For CS‐4OST, 2 mM DTT was added to the reaction mixture. For GalNAc4S‐6OST, 3 mg CS‐A was used instead of chondroitin as the substrate. Reactions were incubated at 37°C. PAP production was quantified via a standard curve, and detection method was performed as described in section 2.4 with a UV wavelength of 260 nm.

The kinetic parameters (*K*_m_ and *k*_cat_) of the recombinant sulfotransferases were determined using a colorimetric assay as previously described (Datta et al. 2020). This assay couples the sulfotransferase reaction with AST IV‐mediated conversion of PAP back to PAPS, accompanied by the release of p‐nitrophenol (PNP) from p‐nitrophenyl sulphate (PNPS), which was monitored spectrophotometrically. The reaction mixture (1 mL) contained 50 mM MES buffer (pH 6.2), 2 mM CaCl_2_, 0.01 mg/mL purified AST IV, 20 mM PNPS, 0.05 mM PAP and varying concentrations of chondroitin substrate (0.1–30 mM). For CS‐4OST, 2 mM DTT was included. Reactions were initiated by the addition of the sulfotransferase and incubated at 37°C. At designated time points, aliquots were withdrawn and mixed with 20 μL stop solution (0.4 M NaOH and 3% SDS, 1:1, v/v) to quench the reaction. PNP formation was measured at 400 nm using a microplate reader. Initial reaction rates (V_0_) were calculated from the linear phase of PNP accumulation. All measurements were performed in triplicate. The V_0_ values at different substrate concentrations were fitted to the Michaelis–Menten equation by nonlinear regression using GraphPad Prism 8.0 to derive *K*_m_ and *k*_cat_.

Enzyme stability was assessed by incubating the enzyme at 4°C and 37°C for 0–5 d. The reaction system contained 50 mM MES buffer (pH 6.2) and 2 mM CaCl_2_. Specifically, the reaction mixture for CS‐4OST was supplemented with 2 mM DTT, 20 μg CS‐0S 9mer and 98 nM PAPS; For CS‐6OST, the mixture contained 20 μg CS‐0S 9mer and 131 nM PAPS. For GalNAc4S‐6OST, 20 μg CS‐A 9mer and 86 nM PAPS was added. After addition of fresh insect cell culture broths with corresponding enzymes, all reactions were incubated at 37°C for 8 h. Oligosaccharide products were detected by HPLC as described in section 2.4 with a detection wavelength of 310 nm. The initial activity (time zero) was set as 100%. Data are presented as the mean ± standard error (SE) of three independent experiments. The stability decay data were fitted to a first‐order kinetic model *A*_t_ = *A*_0_ × *e* ^−kt^, where *A*_t_ is the residual activity at time *t*, *A*_0_ is the initial activity and *k* is the inactivation rate constant. The half‐life (half‐time) *t*_1/2_ was calculated as *t*_1/2_ = ln(2)/*k* (Küchler et al. 2023).

### 2.6 Synthesis of CS Oligosaccharides With Defined Structure

Chondroitin backbone oligosaccharides were enzymatically synthesized using KfoC, UDP‐glucuronic acid (UDP‐GlcA) and UDP‐N‐acetylgalactosamine (UDP‐GalNAc) as described previously (Li et al. 2020). PAPS was prepared from ATP using ATP sulfurylase (ATPS), APS kinase (APSK) and pyrophosphatase (PPA) as described previously (Zhou et al. 2011).

The synthesis and analysis of CS oligosaccharides by CS‐4OST, CS‐6OST and GalNAc4S‐6OST were performed according to the previous publications (Li et al. 2017, 2020). Briefly, for each reaction, 10 mg of CS‐0S 9mer was incubated with 2 mL (735 mU) of CS‐4OST cell culture to produce CS‐A 9mer, or with 1 mL (390 mU) of CS‐6OST cell culture to produce CS‐C 9mer. Additionally, 8 mL of GalNAc4S‐6OST concentrated enzyme was used to generate CS‐E 9mer from 8 mg of CS‐A 9mer. Accurate molecular weights were measured by a QToF mass spectrometer (Waters Xevo G2 XS QToF) with negative ion mode (ESI^−^) in combination with an ACQUITY UPLC I‐Class system.

### 2.7 Synthesis of CS Polysaccharides

CS‐A and C polysaccharides were synthesized from chondroitin polysaccharide substrate using CS‐4OST and CS‐6OST, respectively, with PAPS as the sulphate donor. The reactions were performed under conditions identical to those used for oligosaccharide synthesis (50 mM MES, pH 6.2, 2 mM CaCl_2_, 37°C, 12–16 h), and scaled up to a final volume proportional to the amount of chondroitin, maintaining a substrate concentration of 1 mg/mL. The system contained corresponding enzyme preparation and PAPS, with the addition of 2 mM DTT specifically for CS‐A synthesis. In a scaled preparation, 310 U of cell culture supernatant expressing CS‐4OST was incubated with 2.12 g of chondroitin polysaccharide to produce CS‐A. Similarly, 1.12 g of chondroitin was used to produce CS‐C with 75 U of supernatant expressing CS‐6OST. The CS products were purified via a DEAE‐Sepharose column, which was eluted by a linear gradient of NaCl (0–1 M) in 2 h. The eluted products were then precipitated with three volumes of ethanol, collected by centrifugation, and lyophilized.

The sulfation pattern and reaction efficiency were determined by 2‐aminoacridone (AMAC)‐derivatized disaccharide analysis. Commercial CS disaccharide standards (Δdi‐0S, Δdi‐4S and Δdi‐6S; Sigma‐Aldrich) were used as references for peak identification. Briefly, the CS samples were digested with chondroitinase ABC (ChABC) to produce the CS disaccharides, which were then labelled by AMAC according to previous reports (Chang et al. 2012). Control experiments using standard mixtures confirmed that the digestion and derivatization procedures were quantitative and unbiased. The AMAC‐derivatized disaccharide was analysed by a C18 reversed‐phase column (ZORBAX SB‐C18, 4.6 × 250 mm) with 50 mM ammonium acetate and methanol as mobile phase. Gradient elution was performed as follows: 25%–40% methanol from 0 to 60 min, 40%–100% methanol from 61 to 70 min and held at 100% methanol from 71 to 80 min. The flow rate was maintained at 0.5 mL/min, and the detection wavelength was set at 255 nm. The proportion of the target sulphated disaccharide was calculated based on its peak area relative to the total area of all disaccharide peaks, indicating the efficiency of the sulfation reaction. The yield of the CS polysaccharides was determined by weighing the purified and lyophilized product directly.

### 2.8 ^1^H NMR Spectroscopy Analysis

The structures of the synthesized CS polysaccharides were further confirmed by ^1^H nuclear magnetic resonance (NMR) spectroscopy. Samples (approximately 10 mg) were lyophilized twice with 99.9% D_2_O and finally dissolved in 500 μL D_2_O. ^1^H NMR spectra were recorded at 298 K on a Bruker Avance III HD 500 MHz spectrometer equipped with a 5 mm BBO probe. Spectra were recorded with 64 k data points over a spectral width of 12 ppm, 16 scans, and a relaxation delay of 2 s. The unsulphated chondroitin (CS‐0S) was used as a reference for signal assignment. The characteristic signals corresponding to sulphated positions were identified by comparison with previously reported chemical shifts for chondroitin sulphate derivatives.

### 2.9 Coupled Chondroitin Sulphate Sulfation and PAPS Regeneration Reaction

The coupled reaction system was constructed by combining the CS‐6OST‐catalysed chondroitin sulphate sulfation with PAPS regeneration mediated by the regenerating enzyme AST IV. Based on the original sulfotransferase reaction conditions, the amount of supplemented PAPS was adjusted: PAPS was initially added at 10% of the substrate molar amount as the sulphate donor, and AST IV was included simultaneously to achieve in situ regeneration of PAPS. The reactions were performed in 50 mM MES buffer (pH 6.2) containing 2 mM CaCl_2_, 20 μg of the corresponding oligosaccharide substrate, and CS‐6OST, followed by incubation at 37°C for 12 h. After the incubation, substrate and product were analysed by HPLC using a ProPac PA1 column. All experiments were repeated three times, and data are presented as the mean ± standard deviation.

## 3 Results

### 3.1 Construction of Chondroitinase‐Free Baculovirus

**FIGURE 1:**
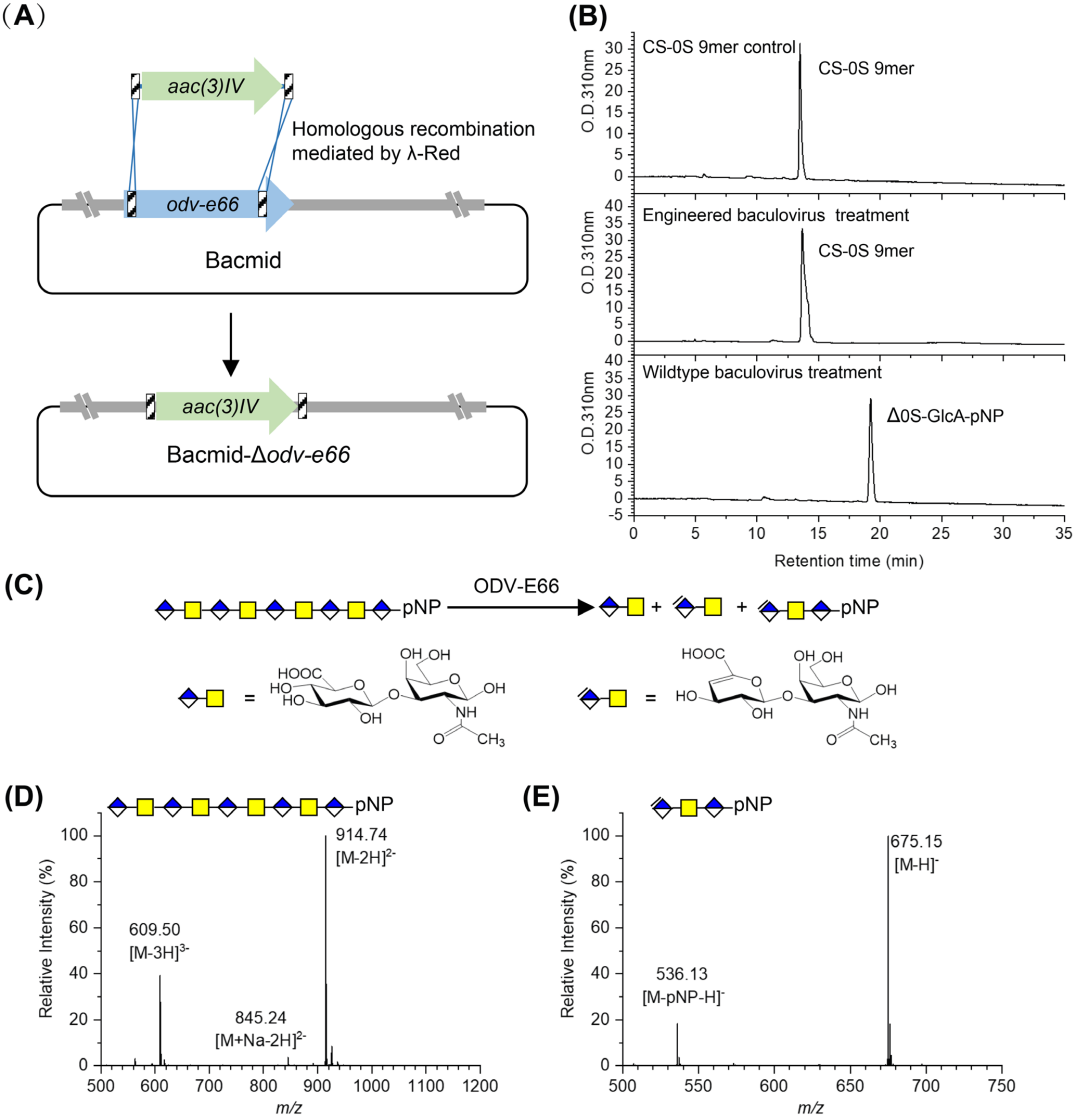
Construction and verification of the *odv‐e66*‐deleted baculovirus. (A) Schematic diagram of the replacement of *odv‐e66* with *aac*(*3*)*IV* via PCR targeting. (B) HPLC analysis of CS‐0S 9mer treated with different insect cell culture broths, including untreated standard CS‐0S 9mer control, treated with engineered baculovirus‐infected *Sf9* cell cultures and treated with wildtype baculovirus‐infected *Sf9* cell cultures. (C) Schematic diagram of CS‐0S 9mer degraded by ODV‐E66. (D) MS analysis of CS‐0S 9mer. (E) MS analysis of Δ0S‐GlcA‐pNP, the degraded product of CS‐0S 9mer.

To inactivate the baculoviral chondroitinase, the segment of the *odv‐e66* gene in the *Autographa californica* multiple nucleopolyhedrovirus (AcMNPV) genome spanning nucleotides 22 to 2056 (encoding amino acid residues Val8 to Gly685, which harbours the chondroitinase domain) was replaced with the apramycin resistance gene *aac(3)IV* via l‐Red‐mediated homologous recombination (Figure 1A). The resulting plasmid, named bacmid‐Δ*odv‐e66*, was verified by PCR (Figure S1) and sequencing. Transfection of insect cell *Sf9* with the correct bacmid‐Δ*odv‐e66* generated the recombinant baculovirus lacking CS degrading activity, which was confirmed by the intact chondroitin nonasaccharide (CS‐0S 9mer) detected after overnight incubation with the virus culture. In contrast, the wildtype baculovirus exhibited potent chondroitinase activity, completely degrading the CS‐0S 9mer into disaccharides, which were confirmed by the HPLC and Mass Spectrometry (MS) analysis (Figure 1B–E).

### 3.2 Expression of Chondroitin Sulfotransferase by the Engineered Baculovirus

**FIGURE 2:**
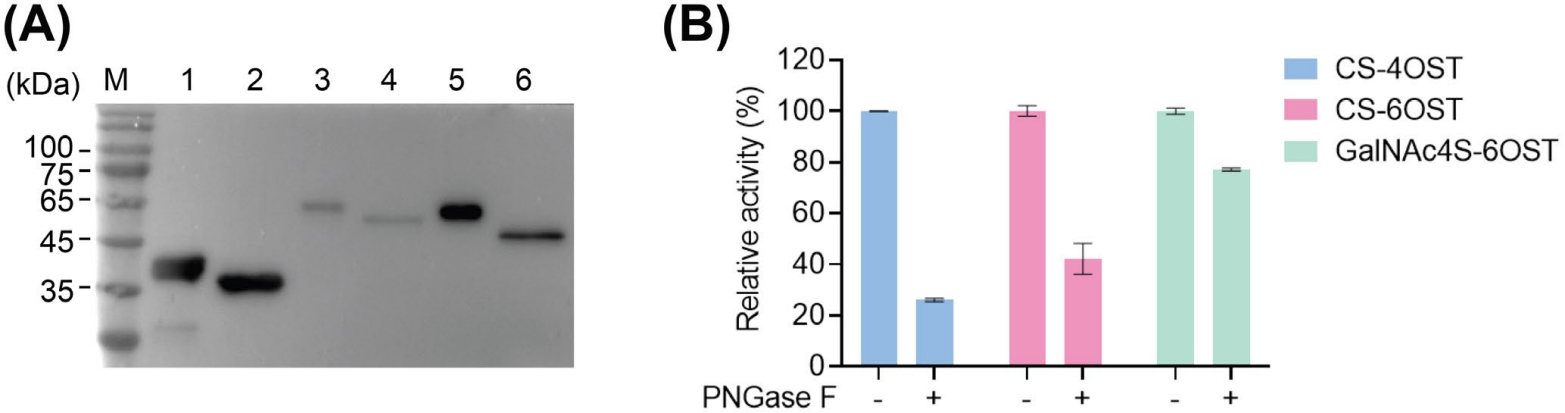
Expression and activity analysis of the recombinant sulfotransferases. (A) Western blot analysis of the recombinant CS‐4OST (42.4 KDa, theoretical), GalNAc4S‐6OST (57.8 KDa, theoretical) and CS‐6OST (54 KDa, theoretical). Lanes 1, 3 and 5 represent the recombinant CS‐4OST, GalNAc4S‐6OST and CS‐6OST secreted into the cell culture supernatant. Lanes 2, 4 and 6 represent the CS‐4OST, GalNAc4S‐6OST and CS‐6OST enzymes after PNGase F treatment, which exhibited a reduced molecular weight due to N‐deglycosylation. (B) Effect of N‐deglycosylation on sulfotransferase activity. Residual activities of CS‐4OST, CS‐6OST and GalNAc4S‐6OST after PNGase F treatment are shown as percentages relative to the untreated control (100%). Data represent mean ± SD from three independent experiments.

Subsequently, chondroitin sulfotransferases CS‐4OST, CS‐6OST and GalNAc4S‐6OST, which were individually fused to the sequence encoding the honeybee melittin signal peptide, were expressed separately in *Sf9* insect cells using the engineered baculovirus‐based Bac‐to‐Bac expression system. Western blot analysis confirmed the successful expression of these recombinant enzymes, which were secreted into the cell culture medium (Figure 2A). Of note, all three proteins showed diffuse bands at molecular weights higher than their predicted sizes. These bands shifted to the expected positions following PNGase F treatment, indicating that these enzymes were N‐glycosylated.

To investigate the role of N‐glycosylation in sulfotransferase function, enzyme activities were compared after deglycosylation mediated by PNGase F. Briefly, recombinant CS‐4OST, CS‐6OST and GalNAc4S‐6OST were incubated with PNGase F at 37°C for 1 h to remove the N‐glycosylation (Figure S2), then their activities were compared with the untreated controls. The results showed that deglycosylation significantly decreased the catalytic activity of these three enzymes to 26%, 42% and 77% of control levels, respectively (Figure 2B), demonstrating that the N‐glycosylation from the baculovirus‐insect cell system is essential for maintaining the activity of chondroitin sulfotransferases.

To further evaluate the enzyme productivity by this engineered expression system, the total sulfotransferase activity in the culture medium was determined by measuring the efficiency of sulphate group transfer from the donor 3′‐phosphoadenosine‐5′‐phosphosulfate (PAPS) to precursor sugar chains. The amount of enzyme required to produce 1 μmol of 3′‐phosphoadenosine 5′‐phosphate (PAP) per minute was defined as one unit (U) of activity. Using chondroitin polymer as the substrate, the enzyme activities of CS‐4OST, CS‐6OST and GalNAc4S‐6OST in the culture broth were determined to be 367.69 ± 16.14 U/L, 388.50 ± 14.36 U/L and 428.48 ± 12.00 U/L, respectively.

### 3.3 Characterization of the Chondroitin Sulfotransferases

The steady‐state kinetic parameters of each sulfotransferase were determined as described in a previous report (Datta et al. 2020). As a result, all these enzymes exhibited high catalytic efficiency, with *K*_m_ values of approximately 0.62 mM, 0.75 mM and 4.95 mM, and corresponding *k*_cat_ values of 3.03, 6.27 and 2.76 s^−1^ for CS‐4OST, CS‐6OST and GalNAc4S‐6OST, respectively (Table 1).

**TABLE 1:** Kinetic analysis of CS‐4OST, CS‐6OST and GalNAc4S‐6OST.

	*K*_m_ (mM)	*k*_cat_ (s^−1^)
CS‐4OST	0.62 ± 0.18	3.03 ± 0.26
CS‐6OST	0.75 ± 0.076	6.27 ± 0.20
GalNAc4S‐6OST	4.95 ± 1.85	2.76 ± 0.49

*Note:* Data are shown as the mean ± standard error (SE) from three independent experiments.

**FIGURE 3:**
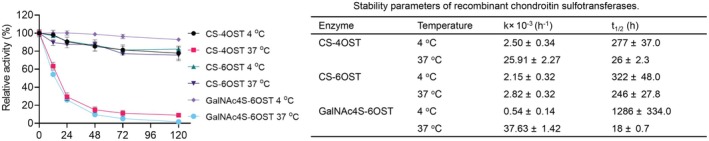
Thermal stability of the recombinant chondroitin sulfotransferases. The stability of the enzymes was assessed at 37°C and 4°C, with the initial activity (time zero) set as 100%. All values are presented as the mean ± standard error (SE) from three independent experiments.

Furthermore, the stability of these recombinant sulfotransferases was evaluated by storing the CS‐4OST, CS‐6OST and GalNAc4S‐6OST at 4°C and 37°C for 5 days, and measuring the residual activity at different time points. CS‐6OST exhibited excellent stability, retaining over 80% of initial activity at 4°C and more than 75% at 37°C after 5 days of incubation. GalNAc4S‐6OST maintained high stability at 4°C, retaining 92% of its activity on day 5. However, its activity decreased sharply at 37°C, dropping to only 1.7% by the end of the 5‐day storage. CS‐4OST retained 77% of its initial activity at 4°C but showed markedly reduced stability at 37°C, with merely 9.0% residual activity remaining after 5 days (Figure 3). Notably, after 12 h of incubation at 37°C, the three enzymes all retained substantial activity, ranging from 54% to 89% of their initial activity. These results indicate that these enzymes possess good stability under refrigerated conditions (4°C), with CS‐6OST proving particularly robust even at physiological temperature (37°C). To further quantify these stability differences, the activity decay profiles were fitted to a first‐order inactivation model (Küchler et al. 2023). The calculated half‐life (*t*_1/2_) confirmed that CS‐6OST was highly stable at both 4°C and 37°C, with values of 322 h and 246 h, respectively (Figure 3). Although GalNAc4S‐6OST underwent rapid inactivation at 37°C (*t*_1/2_ ≈18 h), it was highly stable at 4°C, with t_1/2_ ≈1286 h.

### 3.4 Enzymatic Synthesis of Homogeneous CS

**FIGURE 4:**
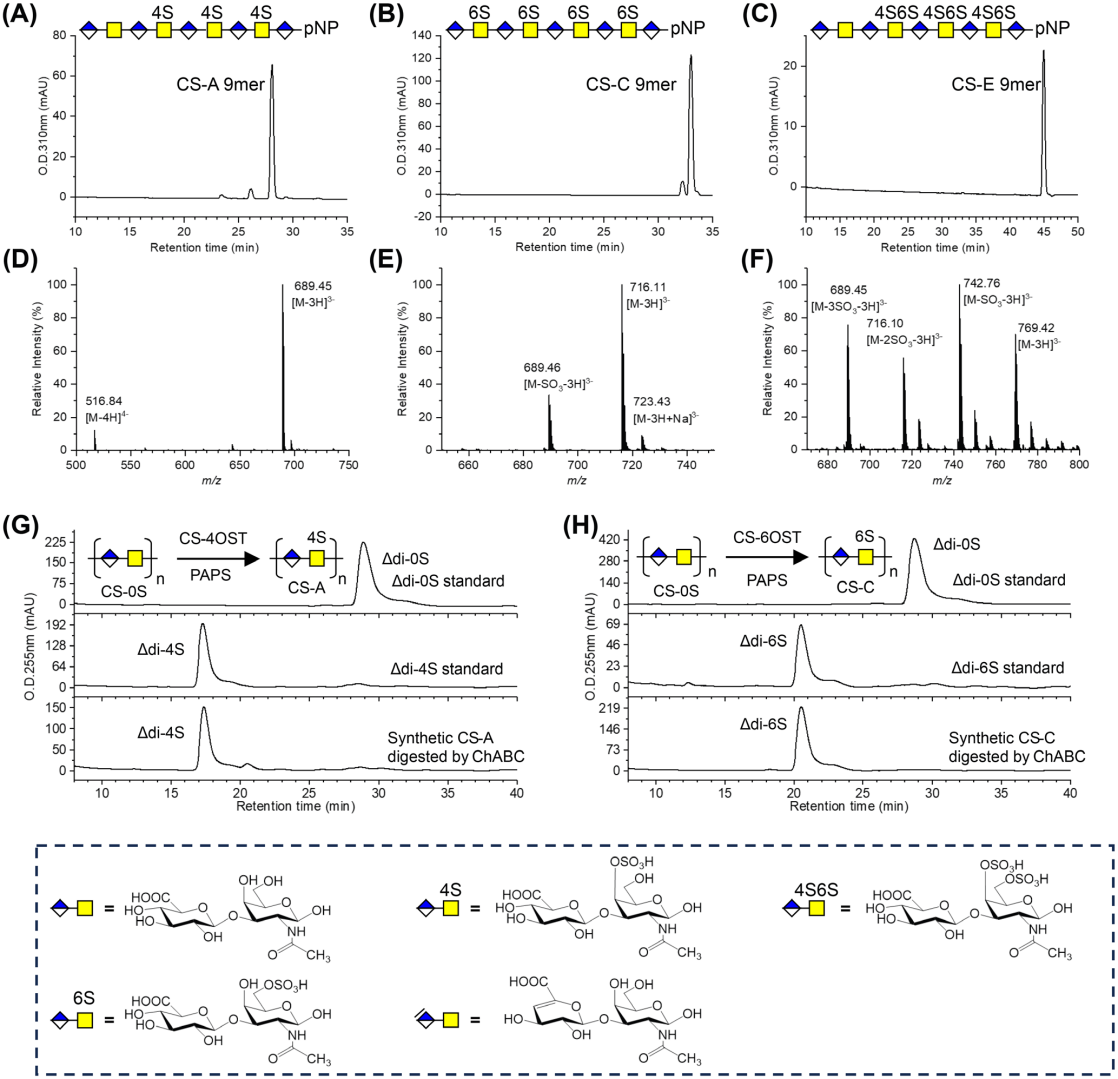
Enzymatic synthesis of CS by the recombinant sulfotransferase expressed by the engineered baculovirus‐insect cell expression system. (A–C) HPLC chromatograms of CS‐A 9mer (A), CS‐C 9mer (B) and CS‐E 9mer (C). (D–F) Negative‐ion mode MS of the synthesized CS‐A 9mer (D), CS‐C 9mer (E) and CS‐E 9mer (F). (G) Schematic workflow and disaccharide composition analysis of synthesized CS‐A. HPLC chromatograms of the unsulphated chondroitin disaccharide standard (Δdi‐0S), CS‐A disaccharide standard (Δdi‐4S) and the enzymatically synthesized CS‐A product digested by ChABC (Δdi‐4S) are shown. (H) Workflow and disaccharide analysis of synthesized CS‐C. HPLC chromatograms of the unsulphated chondroitin disaccharide standard (Δdi‐0S), CS‐C disaccharide standard (Δdi‐6S) and the synthesized CS‐C product digested by ChABC (Δdi‐6S) are indicated.

Owing to the high activity and stability of the recombinant sulfotransferases, as well as the absence of chondroitinase in the engineered expression system, the recombinant chondroitin sulfotransferases expressed in the engineered baculovirus‐insect cell system were directly subjected to CS synthesis. Compared with the cell culture expressing chondroitin sulfotransferase via the wildtype Bac‐to‐Bac expression system, which resulted in the degradation of chondroitin oligosaccharides due to the presence of chondroitinase (Figure S3 and Figure S4), the cell culture containing recombinant sulfotransferase expressed by the engineered baculovirus can efficiently catalyse the sulfation of chondroitin to synthesize CS. Specifically, in the presence of PAPS, the cell culture harbouring CS‐4OST expressed via the engineered baculovirus converted chondroitin nonasaccharide (CS‐0S 9mer) to CS‐A 9mer, with 4‐*O*‐sulfation at GalNAc residues. Similarly, the cell culture containing CS‐6OST successfully sulphated CS‐0S 9mer to CS‐C 9mer, which is characterized by 6‐*O*‐sulfation at GalNAc residues. GalNAc4S‐6OST enzyme catalysed the conversion of CS‐A 9mer to CS‐E 9mer, which bears both 4‐*O*‐ and 6‐*O*‐sulfation on GalNAc residues. All the products were verified by HPLC and MS analysis (Figure 4A–F).

In addition to the synthesis of CS oligosaccharides, these cell culture supernatants harbouring chondroitin sulfotransferases were further used for the sulfation of chondroitin polysaccharide, yielding CS products with homogeneous sulfation patterns. The structures and sulfation homogeneity of the resulting polysaccharides were characterized by disaccharide composition analysis and nuclear magnetic resonance (NMR) spectroscopy. Disaccharide composition analysis revealed that chondroitin sulphate A polysaccharide (CS‐A) produced by CS‐4OST cell culture supernatant contained 96.7% CS‐A disaccharide (Δdi‐4S) and 3.3% chondroitin disaccharide (Δdi‐0S). After purification and dialysis, approximately 2.32 g of CS‐A polysaccharides was obtained from 2.12 g of chondroitin polysaccharide, with a recovery rate of ~90% (Figure 4G). Similarly, chondroitin sulphate C (CS‐C) synthesized by CS‐6OST culture supernatant with 96% CS‐C disaccharide content (Δdi‐6S) (Figure 4H). From 1.12 g of chondroitin polysaccharide, 1.28 g of purified CS‐C polysaccharide was obtained, with a 94% recovery rate. ^1^H NMR spectra of the CS polysaccharides were recorded at 500 MHz in D_2_O. Compared with the unmodified chondroitin (CS‐0S) control, which exhibited a characteristic N‐acetyl proton signal of GalNAc at δ 1.92–1.96 ppm on the ^1^H NMR spectrum, the 4‐*O*‐sulfation of GalNAc (CS‐A) resulted in a characteristic downfield shift of H‐4 (δ ~4.68 ppm), while 6‐*O*‐sulfation (CS‐C) led to diagnostic shifts of H‐6 (δ ~4.16 ppm), confirming the successful formation of the expected CS‐A and CS‐C products (Figures S5–S7). Overall, the chondroitin sulfotransferase expressed in this engineered baculovirus expression system enabled efficient and potentially scalable synthesis of non‐animal‐derived chondroitin sulphate, and the structural characterization confirmed the successful preparation of well‐defined CS‐A and CS‐C polysaccharides with uniform sulfation patterns.

**FIGURE 5:**
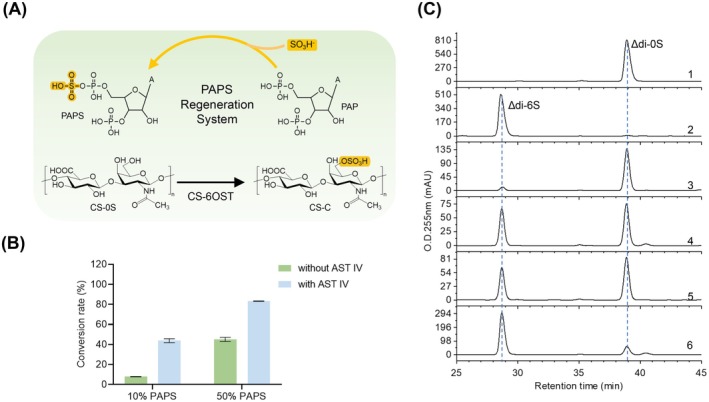
Coupled chondroitin sulphate sulfation and PAPS regeneration system. (A) Schematic diagram of the coupled system for chondroitin sulphate sulfation and PAPS regeneration. The system couples CS‐6OST‐catalysed chondroitin sulphate sulfation with AST IV‐mediated PAPS regeneration, forming a cyclic reaction network. (B) Conversion rates of sulfation reactions under different PAPS proportions (10% and 50%) with or without the PAPS regeneration system (AST IV). Green bars represent reactions without AST IV, and blue bars represent reactions with AST IV. All data represent the mean ± standard deviation of three independent experiments. (C) HPLC chromatograms of sulfation reaction products. All reactions contain CS‐0S and PNPS with varying concentrations of PAPS, with the presence or absence of AST IV and CS‐6OST. Specifically: (1) without CS‐6OST and AST IV, with 100% PAPS; (2) with 100% PAPS and CS‐6OST, without AST IV; (3) with 10% PAPS and CS‐6OST, without AST IV; (4) with 10% PAPS, AST IV and CS‐6OST; (5) with 50% PAPS and CS‐6OST, without AST IV; (6) with 50% PAPS, AST IV and CS‐6OST.

Besides eliminating the need for protein purification and thus reducing purification‐related costs, coupling the PAPS regeneration system with the enzymatic synthesis of CS could further improve the economic efficiency by minimizing consumption of the expensive PAPS. To evaluate the effect of this integrated system on catalytic efficiency, the AST IV mediated PAPS regeneration system was introduced into CS‐6OST‐catalysed sulfation reaction. This coupling strategy led to a significant improvement in sulfation efficiency (Figure 5A). When 50% PAPS was used for sulfation, 42.7% of the substrate was sulphated. Upon coupling with the PAPS regeneration system, the sulfation efficiency increased to 83.1% (Figure 5B,C). When 10% PAPS was used, the conversion reached 42.6% after introducing the AST IV‐mediated PAPS regeneration system, which was 5.5 times higher than that without the regeneration system (7.9% sulfation), representing a remarkable improvement in catalytic efficiency compared with the control reaction.

## 4 Discussion

The baculovirus‐insect cell expression system combines the high efficiency of prokaryotic cells with the capacity for complex post‐translational modifications of mammalian systems, making it a valuable platform for producing animal‐derived proteins. However, the presence of endogenous viral chondroitinase has severely hindered its application in the heterologous expression of chondroitin sulfotransferases. To overcome this limitation, we constructed a chondroitinase‐free baculovirus expression system by deletion of the *odv‐e66* gene. Using the engineered baculovirus, chondroitin sulfotransferases including CS‐4OST, CS‐6OST and GalNAc4S‐6OST were efficiently expressed in *Sf9* insect cells and exhibited high catalytic activity. The removal of chondroitinase contamination enables the direct use of culture supernatant containing secreted sulfotransferases for CS synthesis, eliminating laborious purification process.

ODV‐E66 is a conserved envelope protein of occlusion‐derived virus in baculoviruses that acts as an endogenous chondroitinase. By degrading chondroitin sulphate proteoglycans in the larval midgut peritrophic membrane, it facilitates oral infection in insect hosts (Sugiura et al. 2011). According to previous studies, deletion of the *odv‐e66* gene severely impairs oral infectivity in larvae, but does not affect viral genome replication, budded virus production or infectivity in cultured insect cells (Xiang et al. 2011). On this basis, we constructed a chondroitinase‐free baculovirus system while maintaining its function as a protein expression host. The recombinant bacmid‐Δ*odv‐e66* was successfully transfected into *Sf9* cells and allowed high‐level expression of three active sulfotransferases. Crude enzyme supernatants prepared from this system supported efficient and reliable synthesis of homogeneous CS‐A and CS‐C polysaccharides without degradation, indicating that the system provides favourable expression levels and enzymatic activity for the target proteins and satisfies the experimental requirements for in vitro synthesis of chondroitin sulphate polysaccharides.

Although yeast expression systems are cost‐effective and support glycosylation, they tend to introduce high‐mannose type N‐glycans, which differ significantly from the complex glycosylation patterns in mammalian systems. In contrast, the baculovirus‐insect cell system provides glycosylation closer to that of mammalian cells while also delivering higher recombinant protein yields than mammalian expression platforms (Yin et al. 2007). These characteristics are crucial for obtaining chondroitin sulfotransferases with high catalytic activity and stability, thereby justifying the selection of insect cell expression system for the present study.

A key advantage of this system is that the elimination of chondroitinase contamination allows the culture medium containing secreted sulfotransferases to be directly employed in CS synthesis. Employing the wildtype Bac‐to‐Bac expression system to produce sulfotransferases requires a time‐consuming two‐step purification process, first using heparin affinity chromatography, and then using cobalt‐based resin purification, to eliminate chondroitinase before catalysis. In contrast, the engineered baculovirus expression system allows the sulfotransferases in the cell culture to be directly used for catalytic reactions without any purification. This approach not only eliminates the risk of chondroitinase contamination but also reduces costs. Using the recombinant CS‐4OST and CS‐6OST, CS‐A and CS‐C polysaccharides were produced at gram‐scale with a sulfation degree of 96%.

While the strategy employed in this study, synthesizing the chondroitin sulphate backbone separately before enzymatic sulfation, appears more cumbersome than a one‐pot approach, this design enables precise control over the sulfation pattern and degree for the synthesis of homogeneous chondroitin sulphate subtypes. It also allows each reaction step to be independently scalable to meet large‐scale production demands. Combined with the elimination of sulfotransferase purification achieved through *odv‐e66* gene knockout, this modular strategy strikes a balance between structural precision and overall synthetic efficiency. Thus, while the one‐pot system excels in precursor integration, the engineered baculovirus expression system provides multiple enzymes for precise modifications, demonstrating significant value in generating homogeneous chondroitin sulphate structures.

To achieve a high degree of sulfation, excess PAPS was used, and a large amount of PAP was generated. As a competitive inhibitor of sulfotransferases, PAP reduces the enzyme catalytic efficiency (Chen et al. 2005). To address this issue, we introduced a PAPS regeneration system. Previous studies have demonstrated that the PAPS regeneration system could convert PAP back to PAPS, thereby alleviating the inhibitory effect and enhancing the catalytic efficiency of CS sulfotransferases (Jin et al. 2020). Consistent with these findings, our results also confirmed that the AST IV‐mediated PAPS regeneration system could improve sulfation efficiency and reduce PAPS consumption, which further enhances the practical application of our engineered baculovirus expression system.

As demonstrated in this study, the recombinant chondroitin sulfotransferases expressed in the engineered baculovirus system were effectively glycosylated and exhibited high catalytic activity, which was sufficient for efficient in vitro synthesis. Notably, deglycosylation experiments confirmed that this modification is essential for maintaining enzymatic activity. Therefore, while the glycan structures may vary, the insect‐cell system provides a functional form of glycosylation for these enzymes.

Moreover, due to the chondroitinase‐free background, using the engineered baculovirus to produce more enzymes associated with CS synthesizing or modifying, for example, dermatan sulphate epimerase and dermatan 4‐*O*‐sulfotransferase (Tykesson et al. 2018), could be advantageous. Hence, future studies should focus on further optimization and engineering of the baculovirus‐insect cell expression system to enhance both synthetic efficiency and subtype diversity of chondroitin sulphates. Overall, the engineered baculovirus‐insect cell expression system is an efficient platform for expression of CS‐modifying enzymes free from chondroitinase contamination. The highly active recombinant CS sulfotransferases enable efficient enzymatic synthesis of CS with homogeneous sulfation, providing high‐purity CS materials for scientific research and biomedical applications.

## 5 Conclusions

In this study, an engineered baculovirus‐insect cell expression platform free of chondroitinase contamination was successfully established by deleting the gene encoding the viral envelope protein ODV‐E66, a protein that can be converted to chondroitinase via protease cleavage and thereby degrade CS. The resulting protein expression system enables efficient synthesis of CS at gram‐scale using crude recombinant CS transferases or even cell cultures, saving laborious and costly purification steps. This platform not only provides a reliable, scalable tool for expressing CS‐modifying enzymes to synthesize animal‐free CS, but also sheds light on the production of CS proteoglycans, a kind of macromolecule that has multiple biological functions yet can only be produced by mammalian cells to date.

## Author Contributions

Junyue Li: methodology, investigation, visualization, writing – original draft. Yuqing Tian: methodology, investigation. Lihe She: methodology, investigation. Zhangliang Liu: methodology, investigation. Yanyan Wang: methodology, investigation, project administration. Yingying Zhou: methodology. Jihui Zhang: writing – review and editing. Leilei Zhu: supervision. Huarong Tan: supervision, writing – review and editing. Jine Li: conceptualization, funding acquisition, project administration, supervision, writing – original draft, writing – review and editing.

## Funding

This work was supported by National Natural Science Foundation of China, 32270055. National Key Research and Development Program of China, 2021YFC2103200.

## Conflicts of Interest

The authors declare no conflicts of interest.

## Supporting information


**Figure S1:** PCR verification of bacmid ‐ *odv‐e66*. (A) Schematic diagram of PCR amplification using wildtype bacmid as the template; (B) Schematic diagram of PCR amplification using bacmid‐Δ*odv‐e66* as the template. (C) PCR verification of the bacmid‐Δ*odv‐e66*. Recombinant bacmid clones 1# and 2# were analysed using different primer combinations to confirm the successful replacement of the *odv‐e66* gene. Lanes 1–3: e66checkF/e66checkR; lanes 4 and 6: e66checkF/Ac‐odv‐de66R; lanes 5 and 7: Ac‐odv‐de66F/e66checkR; lanes 8 and 9: Ac‐odv‐de66F/Ac‐odv‐de66R. Template DNA: lane 1, wildtype bacmid (AcODV); lanes 2,4,5,8, clone 1#; lanes 3,6,7,9, clone 2#. M, 1 kb ladder. All the PCR products were of the expected sizes.
**Figure S2:** Western blot analysis of CS‐4OST, CS‐6OST and GalNAc4S‐6OST after PNGase F digestion. Recombinant CS‐4OST, CS‐6OST and GalNAc4S‐6OST were incubated with or without PNGase F and analysed by Western blot using an anti‐His antibody. Lanes 1, 3 and 5 represent untreated CS‐4OST, CS‐6OST and GalNAc4S‐6OST, respectively. Lanes 2, 4 and 6 represent the corresponding enzymes after PNGase F treatment.
**Figure S3:** The HPLC chromatogram of the digested product of CS‐0S 9mer.
**Figure S4:** The ESI‐MS spectrum of the digested product of CS‐0S 9mer.
**Figure S5:** ^1^H NMR spectrum of CS‐0S.
**Figure S6:** ^1^H NMR spectrum of the synthesized CS‐A.
**Figure S7:** ^1^H NMR spectrum of the synthesized CS‐C.
**Table S1:** The primers used in this study.

## Data Availability

The data that support the findings of this study are available from the corresponding author upon reasonable request.
