# Supplementary material for: Engineering Chondroitinase‐Free Baculovirus‐Insect Cell Expression System for Efficient Synthesis of Chondroitin Sulphates
Source: Microb Biotechnol. 2026 May 15;19(5):e70365. doi: 10.1111/1751-7915.70365 (PMC13176938; doi:10.1111/1751-7915.70365)
Supplement: Supplementary file 1 — Figure S1: PCR verification of bacmid ‐ odv‐e66. (A) Schematic diagram of PCR amplification using wildtype bacmid as the template; (B) Schematic diagram of PCR amplification using bacmid‐Δodv‐e66 as the template. (C) PCR verification of the bacmid‐Δodv‐e66. Recombinant bacmid clones 1# and 2# were analysed using different primer combinations to confirm the successful replacement of the odv‐e66 gene. Lanes 1–3: e66checkF/e66checkR; lanes 4 and 6: e66checkF/Ac‐odv‐de66R; lanes 5 and 7: Ac‐odv‐de66F/e66checkR; lanes 8 and 9: Ac‐odv‐de66F/Ac‐odv‐de66R. Template DNA: lane 1, wildtype bacmid (AcODV); lanes 2,4,5,8, clone 1#; lanes 3,6,7,9, clone 2#. M, 1 kb ladder. All the PCR products were of the expected sizes. Figure S2: Western blot analysis of CS‐4OST, CS‐6OST and GalNAc4S‐6OST after PNGase F digestion. Recombinant CS‐4OST, CS‐6OST and GalNAc4S‐6OST were incubated with or without PNGase F and analysed by Western blot using an anti‐His antibody. Lanes 1, 3 and 5 represent untreated CS‐4OST, CS‐6OST and GalNAc4S‐6OST, respectively. Lanes 2, 4 and 6 represent the corresponding enzymes after PNGase F treatment. Figure S3: The HPLC chromatogram of the digested product of CS‐0S 9mer. Figure S4: The ESI‐MS spectrum of the digested product of CS‐0S 9mer. Figure S5: 1H NMR spectrum of CS‐0S. Figure S6: 1H NMR spectrum of the synthesized CS‐A. Figure S7: 1H NMR spectrum of the synthesized CS‐C. Table S1: The primers used in this study. [file MBT2-19-e70365-s001.docx]

Engineering chondroitinase-free baculovirus-insect cell expression system for efficient synthesis of chondroitin sulfates

Junyue Li^1, 2^, Yuqing Tian^1^, Lihe She^1^, Zhangliang Liu^1, 2^, Yingying Zhou^3^, Yanyan Wang^1^, Jihui Zhang^1^, Leilei Zhu^3^, Huarong Tan^1^, Jine Li^1, 2*^

^1^ State Key Laboratory of Microbial Diversity and Innovative Utilization, Institute of Microbiology, Chinese Academy of Sciences, Beijing 100101, China.

^2^ College of Life Sciences, University of Chinese Academy of Sciences, Beijing 100049, China.

^3^ State Key Laboratory of Engineering Biology for Low-Carbon Manufacturing, Tianjin Institute of Industrial Biotechnology, Chinese Academy of Sciences, Tianjin 300308, China.

^*^Corresponding author. Email: lijine@im.ac.cn

**The file includes:**

FIGURE S1…………………………………………………………………………..……………3

FIGURE S2……………………………………………………………………………..…………4

FIGURE S3……………………………………………………………………………..…………5

FIGURE S4……………………………………………………………………………..…………6

FIGURE S5……………………………………………………………………………..…………7

FIGURE S6……………………………………………………………………………..…………8

FIGURE S7……………………………………………………………………………..…………9

Table S1……………………………………………………………… ………………….……...10

**FIGURE S1.** PCR verification of bacmid-Δ*odv-e66*. (A) Schematic diagram of PCR amplification using wildtype bacmid as the template. (B) Schematic diagram of PCR amplification using bacmid-Δ*odv-e66* as the template. (C) PCR verification of the bacmid-Δ*odv-e66*. Recombinant bacmid clones 1# and 2# were analysed using different primer combinations to confirm the successful replacement of the *odv-e66* gene. Lanes 1–3: e66checkF/e66checkR; lanes 4 and 6: e66checkF/Ac-odv-de66R; lanes 5 and 7: Ac-odv-de66F/e66checkR; lanes 8 and 9: Ac-odv-de66F/Ac-odv-de66R. Template DNA: lane 1, wildtype bacmid (AcODV); lanes 2, 4, 5 and 8, clone 1#; lanes 3, 6, 7 and 9, clone 2#. M, 1 kb DNA ladder. All PCR products were of the expected sizes.

**FIGURE S2.** Western blot analysis of CS-4OST, CS-6OST and GalNAc4S-6OST after PNGase F digestion. Recombinant CS-4OST, CS-6OST, and GalNAc4S-6OST were incubated with or without PNGase F and analyzed by Western blot using an anti-His antibody. Lanes 1, 3, and 5 represent untreated CS-4OST, CS-6OST, and GalNAc4S-6OST, respectively. Lanes 2, 4, and 6 represent the corresponding enzymes after PNGase F treatment.

**FIGURE S3.** The HPLC chromatogram of the digested product of CS-0S 9mer.

**FIGURE S4.** The ESI-MS spectrum of the digested product of CS-0S 9mer.

**FIGURE S5.** ^1^H NMR spectrum of CS-0S.

**FIGURE S6.** ^1^H NMR spectrum of the synthesized CS-A.

**FIGURE S7.** ^1^H NMR spectrum of the synthesized CS-C.

**Table S1: The primers used in this study.**

| Primer Name | Sequence (5' to 3') |
| --- | --- |
| Ac-odv-de66F | TAATAAGCAACATTCGACATGTCTATCGTATTGATTATTGCGTACGGCCCACAGAATGA |
| Ac-odv-de66R | ATTGTAAATCAAACGTGCTGTTGTTCAATTTGAAACCGTGGAATAGGAACTTATGAGCT |
| e66check-F | ACTGAATCGGTGTTGGCGTC |
| e66check-R | CGTGATTTGTACGCGTTAGC |
